# Determinants of Antibacterial Activity in *Humulus lupulus* Hop Extracts: Phytochemical Profiling of Prenylated Acylphloroglucinols and Efficacy Against Methicillin-Resistant *Staphylococcus aureus* and Multidrug-Resistant Uropathogenic *Escherichia coli*

**DOI:** 10.3390/ijms27188364

**Published:** 2026-09-19

**Authors:** Przemysław Leszczyński, Dorota Wojnicz, Dorota Tichaczek-Goska, Michał Gleńsk, Alan Gasiński, Joanna Kawa-Rygielska

**Affiliations:** 1 Department of Fermentation and Cereals Technology, Faculty of Biotechnology and Food Science, Wroclaw University of Environmental and Life Sciences, Chełmońskiego 37 Street, 51-630 Wroclaw, Poland; alan.gasinski@upwr.edu.pl (A.G.); joanna.kawa-rygielska@upwr.edu.pl (J.K.-R.); 2 Department of Biology and Medical Parasitology, Faculty of Medicine, Wroclaw Medical University, Mikulicza-Radeckiego 9, 50-345 Wroclaw, Poland; dorota.wojnicz@umw.edu.pl; 3 Department of Pharmacognosy and Herbal Medicines, Wroclaw Medical University, Borowska 211a, 50-556 Wroclaw, Poland; michal.glensk@umw.edu.pl

**Keywords:** *Humulus lupulus* hop extracts, extraction methods, α-acids, β-acids, antibacterial activity, multidrug-resistant bacteria, methicillin-resistant *Staphylococcus aureus*, uropathogenic *Escherichia coli*, supercritical CO_2_ extraction, natural antimicrobials

## Abstract

Optimizing hop (*Humulus lupulus* L.) extraction protocols for maximum antibacterial efficacy remains an open methodological challenge in developing plant-based natural antimicrobials. We compared five extraction protocols—hot ethanol, cold ethanol, espresso/steam, hot water, and cold water—applied to four hop varieties (Citra, Lubelski, Magnat and Marynka), with a commercial supercritical CO_2_ extract as a reference preparation. We characterized extracts by HPLC (α-acids, β-acids, iso-α-acids) and GC-MS (volatile profile of cold ethanol extracts) and determined antibacterial activity as minimum inhibitory concentration (MIC) against reference and clinically relevant multidrug-resistant (MDR) strains of *Escherichia coli* and *Staphylococcus aureus*. Friedman tests confirmed a statistically significant effect of extraction method on MIC values (*p* ≤ 0.004 for *E. coli* ATCC 25922, *S. aureus* ATCC 29213, and clinical MRSA). Ethanol-based protocols consistently produced the highest hop acid concentrations and the lowest MICs against *S. aureus* ATCC 29213 (0.125–0.250 mg/mL) and against clinical MRSA (as low as 0.016 mg/mL), compared with 1.0–15.0 mg/mL for aqueous and steam-based extracts. Clinical MRSA demonstrated increased susceptibility to selected ethanol extracts and to the CO_2_ reference. MDR uropathogenic *E. coli* (UPEC) maintained high-level resistance (MIC 15 mg/mL) to all preparations except the CO_2_ reference (MIC 6 mg/mL). Spearman correlation identified α-acid content as the dominant predictor of antibacterial activity (ρ = −0.927, *p* < 0.001 for *S. aureus* ATCC 29213). These results suggest ethanol-based and supercritical CO_2_ extraction as optimal protocols for producing hop extracts with antibacterial properties targeting Gram-positive pathogens, including MRSA, and provide a rationale for their use as natural antimicrobials.

## 1. Introduction

Antimicrobial resistance (AMR) is one of the most severe challenges in modern medicine and food safety. It is estimated that multidrug-resistant (MDR) bacterial infections caused 1.27 million deaths worldwide in 2019 [1]. Therefore, the need for novel, naturally derived antimicrobial agents has intensified [2,3], and plant-derived extracts, particularly those with established safety profiles, represent a promising and underexplored resources.

Hops (*Humulus lupulus* L.), traditionally utilized in brewing, contain prenylated acylphloroglucinols, specifically α-acids (humulone, cohumulone, adhumulone) and β-acids (lupulone, colupulone, adlupulone), which exhibit a broad spectrum of antibacterial activity [4,5]. While hop compounds have demonstrated efficacy against Gram-positive bacteria, including MRSA, and select Gram-negative pathogens [6,7], the systematic optimization of extraction protocols to maximize the yield of these molecular antibacterial effectors and their efficacy against clinically relevant MDR pathogens remains poorly understood [8,9]. Recent activity-guided fractionation studies have identified β-acids, particularly lupulone and colupulone, as the primary antibacterial constituents in hop extracts, with minimum inhibitory concentrations (MICs) as low as 0.98 µg/mL against certain bacterial strains [10]. This finding underscores the importance of selecting an appropriate extraction method to determine the bioactive chemical composition of hop-derived preparations for antimicrobial applications [2]. In their undissociated (protonated) forms, α- and β-acids partition into bacterial lipid bilayers due to their lipophilic prenyl substituents, functioning as proton ionophores that dissipate the proton motive force—the electrochemical gradient Gram-positive bacteria critically depend upon for ATP synthesis, active nutrient transport, and regulation of intracellular pH [11,12]. The selective activity of hop bitter acids against Gram-positive over Gram-negative bacteria reflects the absence of an outer lipopolysaccharide membrane in the former, which in Gram-negative species constitutes a critical molecular permeability barrier [13].

Despite the recognized antibacterial potential of hop-derived compounds, the relationship between extraction method, compound composition, and antimicrobial outcome has not been systematically evaluated. Extraction method selection profoundly influences the yield, composition, and consequent bioactivity of hop extracts [14,15]: conventional ethanol- and water-based protocols are widely used due to their simplicity and scalability [14,15], while supercritical fluid extraction (SFE) offers superior selectivity for lipophilic prenylated compounds, solvent-free operation, and preservation of thermolabile bioactive molecules [16,17]. A modified two-step SFE protocol applied to the Polish Marynka hop variety produced extracts enriched in α-acids, β-acids, and terpenes, with the crude extract demonstrating superior antibacterial and synergistic effects compared to isolated xanthohumol [18]. Nevertheless, several critical knowledge gaps remain. First, the relative contributions of α-acids, β-acids, and iso-α-acids to antibacterial activity against clinically relevant MDR pathogens have not been systematically quantified across extraction methods. Second, the efficacy of hop extracts against uropathogenic *E. coli*, a leading cause of urinary tract infections and a major reservoir of antibiotic resistance genes, remains poorly characterized [19]. Third, the effects of hop variety and extraction protocol on volatile compound profiles and their potential contribution to overall antimicrobial activity have not been comprehensively evaluated [20].

To address these gaps, the present study systematically evaluated five extraction protocols (hot ethanol, cold ethanol, espresso/steam, hot water and cold water) across four hop varieties—three Polish (Lubelski, Magnat and Marynka) and one American (Citra)—representing a range of α- and β-acid contents. A commercial supercritical CO_2_ extract from the Marynka variety was used as a reference. We quantified α-acid, β-acid, and iso-α-acid content by high-performance liquid chromatography (HPLC), characterized volatile profiles by gas chromatography–mass spectrometry (GC-MS), and determined MICs against reference and clinical MDR strains of *E. coli* and *S. aureus.* Kruskal–Wallis test, Spearman correlation analysis and Friedman test were employed to assess the relationship between extract composition, extraction method, and antibacterial activity. These findings provide a molecular basis for the rational optimization of hop extraction protocols for antibacterial applications targeting clinically relevant MDR pathogens.

## 2. Results

### 2.1. Extraction Yields

Extraction yields varied substantially among the methods and varieties (Table 1). Cold ethanol extraction produced consistently high yields (1680–2050 mg per 10 g of hop pellets). The highest single yield was observed for the Citra espresso/steam extract (2520 mg), although this method showed greater variability across varieties (1465–2520 mg). Hot ethanol extraction yielded 1450–1700 mg, whereas aqueous extractions produced lower yields (hot water: 970–1190 mg; cold water: 919–1046 mg). These differences reflect the superior solubility of hop resins and bitter acids in ethanol compared with that in water.

### 2.2. HPLC Quantification of α-Acids, β-Acids, and Iso-α-Acids

**Figure 1:**
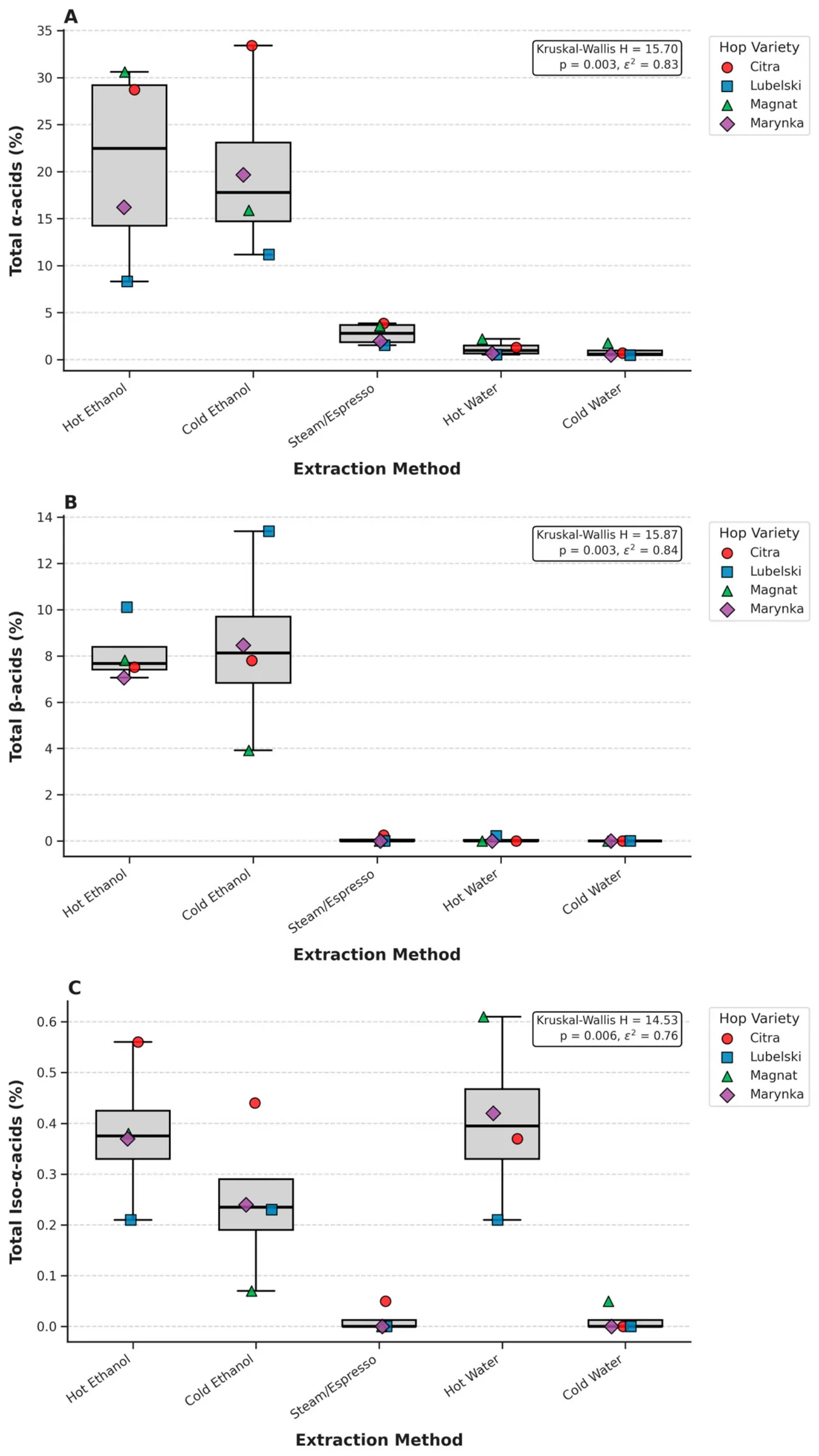
Effect of different extraction methods on the yield of hop bitter acids. Box plots illustrating the percentage content of (**A**) total α-acids, (**B**) total β-acids, and (**C**) total iso-α-acids. Statistical significance was assessed using the Kruskal–Wallis test.

HPLC analysis revealed marked differences in α-acid, β-acid, and iso-α-acid content across extraction methods (Table 1, Figure 1). Visual comparisons highlight the varying extraction efficiencies for the key resin components. Ethanol extractions yielded significantly higher total α-acid content (hot ethanol: median 22.48%, range 8.32–30.62%; cold ethanol: median 17.77%, range 11.17–33.42%) compared to aqueous methods (hot water: median 1.17%, range 0.52–2.20%; cold water: median 0.60%, range 0.46–1.76%) and espresso/steam extraction (median 2.75%, range 1.53–3.86%). The supercritical CO_2_ extract of Marynka exhibited the highest α-acid content (43.47 ± 1.12%). Kruskal–Wallis analysis confirmed that the extraction method was the dominant factor influencing α-acid content (H = 15.70, *p* = 0.003, ε^2^ = 0.83) (Figure 1A).

The β-acid content followed a similar pattern, with hot ethanol (median 7.70%, range 7.06–10.10%) and cold ethanol (median 8.13%, range 3.92–13.39%) extractions yielding substantially higher β-acid levels than aqueous methods (hot water: median 0.11%, range 0.00–0.22%; cold water: median 0.00%) and espresso/steam extraction (median 0.13%, range 0.00–0.26%). The supercritical CO_2_ extract exhibited the highest β-acid content (15.02 ± 0.49%). Kruskal–Wallis analysis confirmed that the extraction method was the dominant factor (H = 15.87, *p* = 0.003, ε^2^ = 0.84) (Figure 1B).

Iso-α-acid content was generally low across all extracts, with hot water (median 0.40%, range 0.21–0.61%) and hot ethanol (median 0.38%, range 0.21–0.56%) extractions yielding the highest levels, likely due to the thermal isomerization of α-acids during extraction. Cold water and espresso/steam extractions produced minimal iso-α-acid content (median 0.00–0.01%). Kruskal–Wallis analysis confirmed that the extraction method was a significant factor (H = 14.53, *p* = 0.006, ε^2^ = 0.76) (Figure 1C).

Among the hop varieties, Citra and Magnat exhibited the highest α-acid content in the ethanol extracts (28.73–33.42% and 15.89–30.62%, respectively), consistent with their declared high α-acid content. Despite its low declared α-acid content (2.7%), Lubelski produced extracts with moderate α-acid levels (8.32–11.17%) and the highest β-acid content (10.10–13.39%), reflecting its high declared β-acid content (4.0%).

**Figure 2:**
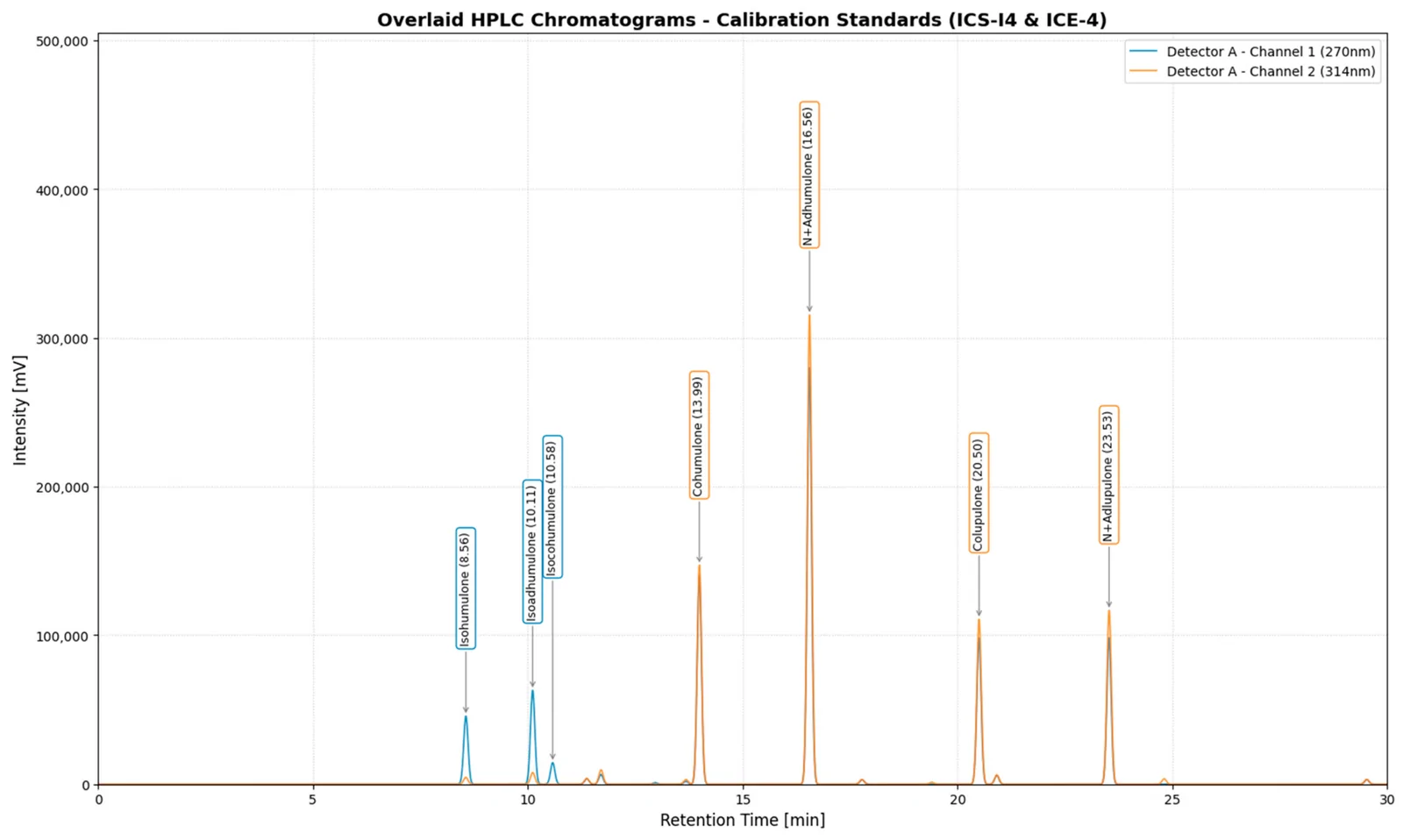
Representative HPLC chromatogram of a mixed reference standard solution (ICE-4 and ICS-I4 certified calibration extracts) illustrating the elution profiles of bitter acids. Peak assignments indicate individual α-acids (humulone, cohumulone, adhumulone), β-acids (lupulone, colupulone, adlupulone), and iso-α-acids (cis/trans-isohumulone, cis/trans-isocohumulone, cis/trans-isoadhumulone) at two detection wavelengths (270 nm and 314 nm).

Representative HPLC chromatogram illustrating the elution profiles of analytical standards is presented in Figure 2. Peak identification in all hop extracts was confirmed by co-elution with certified reference standards (ICE-4 [21] and ICS-I4 [22]) and by comparison of UV absorption spectra recorded using a photodiode array detector.

### 2.3. GC-MS Analysis of Volatile Compounds

GC-MS analysis of unconcentrated cold ethanol hop extracts identified 20 volatile compounds with a mass spectral similarity index ≥ 90% (NIST 17 library), including monoterpenes, sesquiterpenes, and oxidized derivatives (Table 2). Compounds detected in blank runs and IS-only control runs were excluded from the dataset prior to quantification. β-Myrcene was the most abundant volatile compound across all varieties (Citra: 1019.71 ± 97.11 µg/mL; Lubelski: 282.70 ± 12.34 µg/mL; Magnat: 1154.99 ± 31.23 µg/mL; Marynka: 845.03 ± 14.80 µg/mL), followed by β-caryophyllene (31.67–174.20 µg/mL) and α-humulene (99.31–303.36 µg/mL). (E)-β-Farnesene was particularly abundant in Marynka (550.00 ± 9.84 µg/mL) and Magnat (136.70 ± 4.65 µg/mL), while Citra exhibited high levels of methyl trans-geranate (67.79 ± 81.56 µg/mL) and linalool (18.25 ± 1.46 µg/mL). Dehydrocohumulinic acid, an oxidation product of cohumulone, was detected in Citra (33.70 ± 2.82 µg/mL), Lubelski (39.83 ± 9.93 µg/mL), and Marynka (34.99 ± 6.39 µg/mL), but not in Magnat. These volatile profiles reflect varietal differences in terpene biosynthesis and may contribute to the overall antimicrobial activity of hop extracts. Compounds were identified on the basis of Kovàts retention indices and mass spectral comparison with the NIST 17 library; similarity indices are reported in Table 2. Notably, GC-MS analysis is presented exclusively for the cold ethanol extracts, as the processing conditions of the other extraction methods evaluated in this study led to the evaporation of sensitive volatile compounds or their poor solubility in water, rendering them undetectable.

### 2.4. Antimicrobial Activity

A comparison of MIC values demonstrated clear differences in antimicrobial activity among the extracts obtained using different extraction methods (Table 3). Ethanol-based extracts exhibited the highest and most consistent activity, particularly against Gram-positive *S. aureus* ATCC 29213 and MRSA. For *S. aureus* ATCC 29213, MICs of hot- and cold-ethanol extracts ranged from 0.125 to 0.250 mg/mL, whereas MICs of steam-assisted and aqueous extracts were substantially higher (2–10 mg/mL). A similar pattern was observed for a clinical MRSA isolate, for which ethanol extracts showed MICs as low as 0.016 mg/mL compared to up to 15 mg/mL for aqueous extracts. Against *E. coli* ATCC 25922, ethanol extracts consistently showed MICs of 4 mg/mL, whereas steam-assisted and aqueous extracts showed MICs of 15 mg/mL. The supercritical CO_2_ extract obtained from the Marynka variety exhibited particularly high activity, with MICs of 4, 6, 0.125, and 0.032 mg/mL against *E. coli* ATCC 25922, clinical *E. coli*, *S. aureus* ATCC 29213, and clinical MRSA, respectively. However, this extraction method was evaluated for only one hop variety and therefore cannot be directly generalized to other extraction methods.

Overall, ethanol extraction yielded the most consistently active extracts, particularly against reference *S. aureus* and MRSA, whereas the aqueous and steam extracts exhibited substantially higher MICs.

### 2.5. Correlation Between Compound Content and Antimicrobial Activity

To further investigate the relationship between extract composition and antibacterial activity, Spearman’s rank correlation analyses were performed between HPLC-quantified hop acid content and MIC values (Table 4). α-Acid content showed a strong inverse correlation with MIC values for *E. coli* ATCC 25922 (ρ = −0.858, *p* < 0.001), *S. aureus* ATCC 29213 (ρ = −0.927, *p* < 0.001), and clinical MRSA (ρ = −0.891, *p* < 0.001). A significant inverse correlation was also observed between β-acid content and MIC values for *E. coli* ATCC 25922 (ρ = −0.671, *p* = 0.024), *S. aureus* ATCC 29213 (ρ = −0.753, *p* = 0.007), and clinical MRSA (ρ = −0.760, *p* = 0.007). In contrast, no significant association was detected between iso-α-acid content and antibacterial activity against any of the tested strains. For clinical *E. coli*, correlation analysis was not applicable because all extracts exhibited the same MIC value of 15 mg/mL.

Extraction yield alone did not consistently reflect antibacterial potency. For example, the Citra espresso/steam extract showed the highest yield among the Citra extracts (2520 mg), but contained only 3.86% α-acids and exhibited an MIC of 2 mg/mL against *S. aureus* ATCC 29213 (Table 1 and Table 3). In contrast, the Citra hot-ethanol extract yielded 1600 mg, contained 28.73% α-acids, and exhibited an MIC of 0.125 mg/mL (Table 1 and Table 3). Thus, the extraction procedure affected not only the amount of recovered material but also the chemical profile of the resulting extract.

A distinct compositional threshold was evident when evaluating extract efficacy: extracts with total α-acid content ≥ 3% *w*/*w* (*n* = 11) achieved low MICs of 0.125–2.000 mg/mL against *S. aureus* ATCC 29213 (median 0.250 mg/mL), whereas those with <3% *w*/*w* (*n* = 10) yielded substantially higher MICs of 4.0–10.0 mg/mL (median 5.0 mg/mL), a 20-fold shift in median potency. High β-acid content (>7% *w*/*w*), observed exclusively in ethanol-based extracts and the commercial supercritical CO_2_ reference (Table 1), was consistently associated with MICs ≤ 0.250 mg/mL against *S. aureus* ATCC 29213 (Table 3), suggesting that β-acids contribute to antibacterial potency when present at concentrations substantially exceeding their quantification threshold.

The extraction procedure also had a significant overall effect on the MIC values for *E. coli* ATCC 25922 (Friedman’s χ^2^ = 16.00, *p* = 0.003), *S. aureus* ATCC 29213 (χ^2^ = 15.78, *p* = 0.003), and clinical MRSA (χ^2^ = 15.28, *p* = 0.004) (Table 4). Nevertheless, a consistent descriptive pattern was observed, with ethanol-derived extracts generally exhibiting lower MIC values than those obtained using water- or steam-based procedures. This pattern was particularly evident for *S. aureus* and MRSA.

**Figure 3:**
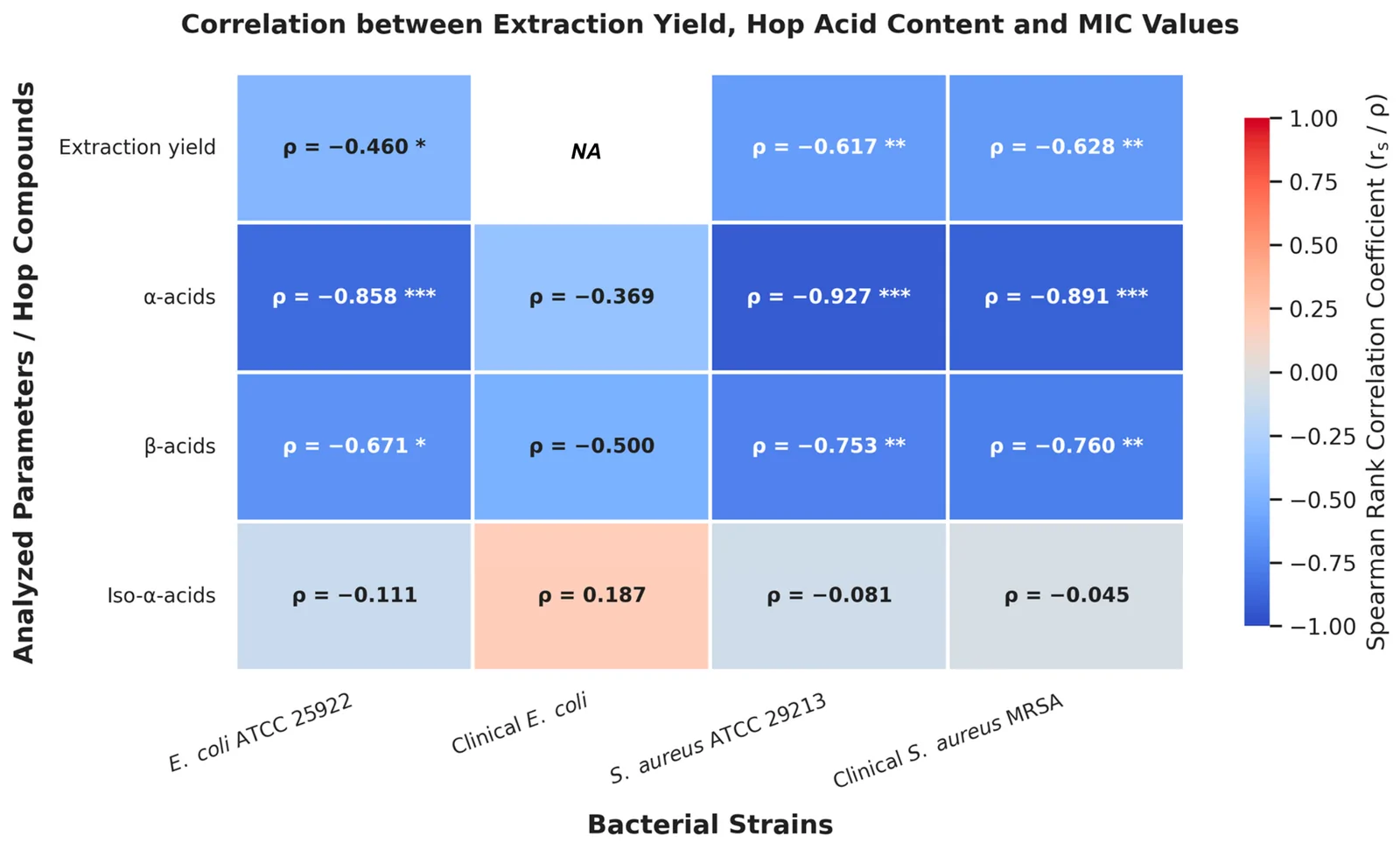
Spearman’s rank correlation heatmap between compound content (% *w*/*w*) or extraction yield (mg) and MIC values. For α-acids and extraction yield, *n* = 21 and 20, respectively; for β-acids, *n* = 11; for iso-α-acids, *n* = 15. *NA*, not applicable because all extracts used against clinical *E. coli* had the same MIC value (15 mg/mL), resulting in zero variance. *p* < 0.05 indicates a statistically significant result. Significance codes: * *p* < 0.05; ** *p* < 0.01; *** *p* < 0.001.

The obtained results indicate the poor sensitivity of Gram-negative bacteria to hop components. Clinical *E. coli* maintained uniform intrinsic resistance across nearly all tested experimental preparations (MIC = 15 mg/mL). Even the highly concentrated commercial CO_2_ reference achieved only a marginally better result (MIC = 6 mg/mL; Table 3), highlighting the known limitations of hop acids against Gram-negative isolates. Because all laboratory preparations yielded an identical MIC value for the clinical *E. coli* isolate, rank-based correlation analysis was not applicable for this strain (Figure 3).

## 3. Discussion

### 3.1. Extraction Method as the Primary Determinant of Bitter Acid Content and Antibacterial Activity

The present study demonstrated that extraction method is the dominant factor influencing the yield, composition, and antibacterial activity of hop extracts. Ethanol-based extractions, particularly cold ethanol, consistently delivered the highest total hop acid concentrations and the lowest MIC values (0.125–0.250 mg/mL against *S. aureus* ATCC 29213 and as low as 0.016 mg/mL against clinical MRSA), reflecting the superior solubility of these lipophilic prenylated acylphloroglucinols in organic solvents [14,15]. In contrast, aqueous methods yielded extracts with α-acid content below 3% *w*/*w* and correspondingly poor antimicrobial efficacy (MIC 4.0–15.0 mg/mL) consistent with the poor water solubility of undissociated α- and β-acids at neutral pH [23]. The espresso/steam method occupied an intermediate position, achieving moderate α-acid extraction (1.5–3.9% *w*/*w*) with correspondingly moderate activity (MIC 1.0–15.0 mg/mL).

Supercritical CO_2_ extraction produced an extract with the highest α-acid (43.47%) and β-acid (15.02%) content, consistent with previous reports demonstrating the efficiency of SFE for recovering hop bitter acids [16,17,18]. The advantages of SFE include selectivity for lipophilic compounds, solvent-free operation, and preservation of thermolabile bioactive compounds [24]. These findings support the use of SFE as a preferred method for producing hop extracts intended for food preservation and nutritional applications. These extraction-driven differences in chemical composition are reflected in the observed antimicrobial activity patterns. Spearman correlation analysis confirmed that α-acid content was the strongest predictor of antibacterial efficacy (ρ = −0.927 for *S. aureus* ATCC 29213; ρ = −0.891 for clinical MRSA), supporting the view that the enrichment of α-acids achieved by ethanol- or SFE-based protocols is a key determinant of extract potency.

The additional correlation analysis provides quantitative support for the association between the chemical composition of hop extracts and their antibacterial potency. Among the hop acid classes quantified by HPLC, α-acids showed the strongest and most consistent inverse association with MIC values, particularly against *S. aureus* ATCC 29213 and clinical MRSA. β-acids were also significantly associated with antibacterial activity, although these associations were less consistent than those observed for α-acids. In contrast, no significant association was observed between iso-α-acid content and MIC values. These findings suggest that the enrichment of α-acids achieved by a specific extraction procedure may be an important factor contributing to the antibacterial potency of the resulting extract.

The results also indicate that extraction yield should not be considered a surrogate measure of antibacterial activity. However, extracts with relatively high mass yields did not necessarily exhibit the greatest antibacterial potency. For example, the Citra espresso/steam extract yielded more material than the corresponding hot-ethanol extract, but contained substantially less α-acids and showed a 16-fold higher MIC against *S. aureus* ATCC 29213. This observation emphasizes that extraction selectivity, rather than extraction yield alone, is relevant when the objective is to obtain extracts enriched with antibacterial constituents.

The Friedman analysis demonstrated an overall effect of the extraction procedure on MIC values for the reference strains and clinical MRSA. Nevertheless, the consistent direction of the observed differences, together with the strong correlations between α-acid content and antibacterial activity, supports the conclusion that ethanol-based extraction produces chemically distinct extracts with greater antibacterial potency.

Importantly, the present findings should not be interpreted as demonstrating that α-acids alone are responsible for the observed antibacterial activity. Hop extracts are complex mixtures containing multiple compounds, and correlations based on extract-level measurements cannot establish causality or exclude synergistic or antagonistic interactions among constituents. Nevertheless, the strong association between α-acid content and antibacterial potency provides evidence that α-acid enrichment is an important compositional characteristic associated with the activity of the extracts. Direct testing of purified individual hop acid compounds would be required to establish their intrinsic antibacterial activity and their individual contribution to the activity of the complex extracts.

It must be acknowledged that the tested preparations represent complex multicomponent mixtures of α-acids, β-acids, iso-α-acids, and other phytochemicals rather than purified individual compounds. Therefore, while the strong correlations observed between α-acid content and MIC values strongly implicate α-acids as primary antimicrobial determinants, synergistic or antagonistic interactions among co-extracted congeners cannot be entirely excluded and warrant further investigation using purified fractions.

### 3.2. Molecular Mechanisms of α-Acid and β-Acid Antibacterial Activity

The strong negative correlations between α-acid content and MIC values against *S. aureus* ATCC 29213 (ρ = −0.927, *p* < 0.001) and clinical MRSA (ρ = −0.891, *p* < 0.001) are consistent with the established ionophoric mechanism of prenylated acylphloroglucinols: the undissociated forms of α- and β-acids partition into the lipid bilayer of Gram-positive bacterial membranes and dissipate the proton motive force, resulting in ATP depletion and broad bactericidal effects [11,12]. The selective activity against Gram-positive but not Gram-negative bacteria observed in the present study reflects the well-known role of the outer membrane as an effective permeability barrier against lipophilic hop acids [13].

The heightened susceptibility of clinical MRSA to certain ethanolic extracts (MIC 0.016 mg/mL) is particularly noteworthy, as it suggests that hop bitter acids may retain their activity against antibiotic-resistant strains. This finding is consistent with recent reports demonstrating that acetone/ethanol hop extracts exhibit antimicrobial activity against MRSA strains comparable to reference antibiotics, with synergistic effects when combined with amikacin and ciprofloxacin. The ability of hop extracts to penetrate and kill biofilm-embedded MRSA cells further supports their potential as adjunctive agents for food-contact sanitization and topical applications [25].

### 3.3. Intrinsic Resistance of Gram-Negative Bacteria: Molecular Basis

The high resistance of both reference *E. coli* ATCC 25922 and clinical MDR UPEC to most hop extracts (MIC 15.0 mg/mL) is consistent with the well-documented intrinsic resistance of Gram-negative bacteria to lipophilic antimicrobials, mediated by the outer membrane barrier and efflux pump systems [13]. Notably, the supercritical CO_2_ extract exhibited moderate activity against the reference *E. coli* ATCC 25922 (MIC 4.0 mg/mL) and clinical UPEC (MIC 6.0 mg/mL), the only extract with measurable activity against the clinical UPEC isolate. This unique activity may reflect the higher concentration of β-acids in the CO_2_ extract, as β-acids have been reported to exhibit greater activity against Gram-negative bacteria compared to α-acids [10,26]. However, the MIC values remain substantially higher than those observed against Gram-positive bacteria, limiting the practical utility of hop extracts for controlling Gram-negative foodborne pathogens.

From a food safety perspective, the potent activity of hop extracts against *S. aureus* and MRSA supports their potential as natural antimicrobials in food preservation where Gram-positive pathogen control is critical [10,27]. Further studies are necessary to translate these findings into practical applications, specifically by addressing formulation, stability, and activity retention within complex food matrices.

### 3.4. Varietal Differences and Implications for Raw Material Selection

Citra and Magnat, high-α-acid varieties, produced ethanolic extracts with the highest α-acid content (28.73–33.42% and 15.89–30.62%, respectively) and potent antibacterial activity against Gram-positive bacteria. Lubelski, a low-α-acid, high-β-acid variety, produced extracts with the highest β-acid content (10.10–13.39%) and exhibited potent activity against clinical MRSA (MIC 0.016 mg/mL for hot ethanol extract). These findings suggest that varietal selection should be guided by the intended application: high-α-acid varieties (Citra, Magnat) are preferred for applications requiring broad-spectrum activity against Gram-positive bacteria, while high-β-acid varieties (Lubelski) may offer advantages for targeting specific resistant strains [10,28].

Seasonal and agronomic factors also influence hop bitter acid content and composition. Seasonal profiling of Cascade leaves revealed humulone and lupulone levels of approximately 0.3 and 0.2 mg/g, respectively, with variations across harvest times [29]. These crop-level variations underscore the importance of raw material characterization and quality control in the production of hop-based functional ingredients.

### 3.5. Volatile Compounds and Potential Contributions to Antimicrobial Activity

GC-MS analysis identified 20 volatile compounds in cold ethanol extracts, including monoterpenes (β-myrcene, limonene, linalool), sesquiterpenes (β-caryophyllene, α-humulene, farnesene), and oxidized derivatives (dehydrocohumulinic acid). Although the antimicrobial activity of hop extracts is primarily attributed to α- and β-acids, certain volatile terpenes have been reported to exhibit antibacterial and antifungal properties [30,31]. β-Caryophyllene and α-humulene, abundant in all varieties tested, have demonstrated antimicrobial activity against Gram-positive bacteria and fungi, potentially contributing to the overall bioactivity of hop extracts [32]. However, the low concentrations of volatile compounds in dried extracts (compared to bitter acids) suggest that their contribution to antibacterial activity is minor under the conditions tested. Future studies employing fractionation and reconstitution experiments are needed to quantify the relative contributions of volatile compounds to the antimicrobial activity of hop extracts.

### 3.6. Limitations and Future Directions

Several limitations of the present study should be acknowledged. First, antimicrobial susceptibility testing of clinical isolates was based on a limited number of strains, with one clinical isolate representing each bacterial group. Although the MRSA and UPEC isolates were confirmed as multidrug-resistant, this limited sample size restricts the generalizability of the findings. Further studies involving a larger and more diverse panel of clinical isolates are needed to assess the variability of susceptibility to hop extracts. Second, the mechanisms underlying the particularly high activity of the supercritical CO_2_ extract against *E. coli* remain unclear. Differences in the composition and relative proportions of bioactive constituents may contribute to this activity, but their individual roles cannot be established from the present data. Further studies involving metabolomic profiling and mechanistic analyses are therefore warranted. Third, the present study focused exclusively on planktonic bacterial cells. Because biofilm formation can increase bacterial tolerance to antimicrobial agents, future studies should evaluate the most promising hop extracts against biofilm-embedded cells using standardized approaches, including Minimum Biofilm Eradication Concentration (MBEC) assays. Such studies would further strengthen the translational relevance of the findings [33]. Another limitation concerns the stability of hop bitter acids under conditions relevant to food processing and storage. Thermal treatment, pH variation, oxidation, and interactions with food matrix components may affect their stability and antimicrobial activity. These factors should therefore be systematically evaluated to guide formulation strategies for food applications [34]. In addition, the commercial supercritical CO_2_ extract was included as an industry-standard reference, but the manufacturer did not disclose the extraction parameters, precluding exact reproducibility. Future studies should use laboratory-scale SFE with fully defined and reported parameters. Future research should also investigate combination strategies involving hop extracts or bitter acids and other natural antimicrobials. Combinations with organic acids, essential oils, or bacteriocins may provide synergistic or additive effects, potentially reducing the required concentrations and undesirable sensory effects [25,35]. Finally, the antimicrobial activity observed under controlled in vitro conditions may not directly translate to complex food or biological matrices. Interactions with matrix components may affect the stability, availability, and efficacy of hop-derived compounds. Therefore, the most promising extracts should be evaluated in relevant food matrices using challenge tests. Where appropriate, subsequent studies should also assess their safety and efficacy in suitable in vivo models to support the practical and regulatory evaluation of hop extracts as natural food preservatives.

## 4. Materials and Methods

### 4.1. Plant Material

T90 hop pellets of four varieties were used in this study: Lubelski (Polish Hops, Karczmiska, Poland; declared α-acid content 2.7% *m*/*m*, declared β-acid content 4.0% *m*/*m*), Marynka (Polish Hops; declared α-acid content 8.2% *m*/*m*, declared β-acid content 2.7% *m*/*m*), Magnat (Polish Hops; declared α-acid content 12.7% *m*/*m*, declared β-acid content 2.8% *m*/*m*), and Citra (Yakima Chief Hops, Yakima, WA, USA; declared α-acid content 12.5% *m*/*m*, declared β-acid content 2.0% *m*/*m*). A commercial supercritical CO_2_ extract of the Marynka variety (Powiśle sp.j., Kępa Chotecka, Poland), with a declared α-acid content of 40% *m*/*m*, soft resin content of 73.6% m/m and hard resin content of 10.5%, was also included. All hop pellets and CO_2_ extract were stored at −20 °C until use to prevent oxidative degradation of bitter acids.

### 4.2. Bacterial Strains

Four bacterial strains representing both reference quality control strains and clinical multidrug-resistant isolates were employed: *Escherichia coli* ATCC 25922 (reference quality control strain, wild-type), clinical *E. coli* (multidrug-resistant, uropathogenic UPEC), *Staphylococcus aureus* ATCC 29213 (reference quality control strain, methicillin-susceptible MSSA), and clinical MRSA (methicillin-resistant *Staphylococcus aureus*) [36]. Clinical strains of uropathogenic *E. coli* and MRSA were obtained from the collection of the Department of Biology and Medical Parasitology, Wroclaw Medical University, Poland. Based on their antimicrobial susceptibility profiles, both strains were classified as multidrug-resistant (MDR) [36]. Reference strains were used as quality control strains for antimicrobial susceptibility testing. All strains were maintained on nutrient agar supplemented with glycerol and stored at −20 °C.

### 4.3. Reagents and Standards

HPLC-grade methanol (≥99.9%, Merck, Darmstadt, Germany), ethanol (96%, analytical grade, Honeywell, Charlotte, NC, USA), orthophosphoric acid (85%, density 1.71 g/cm^3^, Sigma-Aldrich, St. Louis, MO, USA), and Class 1 water (ISO 3696 [37]) prepared using a Milli-Q system (Merck Millipore, Burlington, MA, USA) were used. ICE-4 reference standard (α-acids and β-acids calibration, Labor Veritas, Zurich, Switzerland) and ICS-I4 reference standard (iso-α-acids calibration, Labor Veritas) were employed for HPLC quantification. Mueller–Hinton broth (MHB; Pol-Aura, Gdańsk, Poland) was used for antimicrobial susceptibility testing.

### 4.4. Extraction Procedures

Five distinct extraction protocols were applied to each hop variety.

#### 4.4.1. Hot Water Extraction

Hop pellets (10.0 g) were placed in a 500 mL round-bottom flask containing deionized water (150 mL). The mixture was heated to 60 °C in a water bath for 2 h. After extraction, the mixture was filtered through a Whatman No. 1 filter paper under vacuum. The filtrate was transferred to a rotary evaporator (Büchi R-210 Rotavapor System with Chiller, Büchi Labortechnik AG, Flawil, Switzerland) and concentrated at 45 °C under reduced pressure (35 mbar). The concentrate was dried in vacuo at 25 °C for 24 h.

#### 4.4.2. Cold Water Extraction

Hop pellets (10.0 g) were placed in a 500 mL round-bottom flask containing deionized water (150 mL). The mixture was stirred at room temperature (20 ± 2 °C) for 24 h. After extraction, the mixture was filtered through Whatman No. 1 filter paper under vacuum. The filtrate was transferred to a rotary evaporator and concentrated at 45 °C under reduced pressure (35 mbar). The concentrate was dried in vacuo at 25 °C for 24 h.

#### 4.4.3. Hot Ethanol Extraction

Hop pellets (10.0 g) were placed in a 500 mL round-bottom flask containing 96% ethanol (150 mL). The mixture was heated to 60 °C in a water bath for 2 h. After extraction, the mixture was filtered through Whatman No. 1 filter paper under a vacuum. The filtrate was transferred to a rotary evaporator and concentrated at 45 °C under reduced pressure (35 mbar). The concentrate was dried in vacuo at 25 °C for 24 h.

#### 4.4.4. Cold Ethanol Extraction

Hop pellets (10.0 g) were placed in a 500 mL round-bottom flask containing 96% ethanol (150 mL). The mixture was stirred at room temperature (20 ± 2 °C) for 24 h. After extraction, the mixture was filtered through Whatman No. 1 filter paper under a vacuum. The filtrate was transferred to a rotary evaporator and concentrated at 45 °C under reduced pressure (35 mbar). The concentrate was dried in vacuo at 25 °C for 24 h.

#### 4.4.5. Espresso/Steam Extraction

Hop pellets (10.0 g) were placed in the portafilter of a household espresso machine (Zelmer type 132012, Rzeszów, Poland). Hot water (approximately 90 °C) was forced through the hop pellets under pressure (approximately 9 bar) to produce 150 mL of the extract. The extract was transferred to a rotary evaporator and concentrated at 45 °C under reduced pressure (35 mbar). The concentrate was dried in vacuo at 25 °C for 24 h.

#### 4.4.6. Supercritical CO_2_ Extraction

A commercial supercritical CO_2_ extract of the Marynka variety (Powiśle sp.j.) was used in this study. The extraction parameters were not disclosed, as they constitute a trade secret of the manufacturer.

All dried extracts were weighed to determine the extraction yield (mg extract per 10 g hop pellets) and stored at −20 °C until further analyses.

### 4.5. High-Performance Liquid Chromatography (HPLC) Analysis

HPLC quantification of α-acids, β-acids, and iso-α-acids was performed using a modified version of the European Brewery Convention methods EBC 7.7 (for sample preparation) and EBC 7.8 (for analysis of Iso-α-, α-, and β-acids in hop and isomerised hop extracts by HPLC) [38].

#### 4.5.1. Sample Preparation

Dried hop extract (50.0 ± 0.5 mg) was accurately weighed into a 15 mL polypropylene centrifuge tube. Acidified methanol (3 mL, containing 2 mL/L orthophosphoric acid) was added, and the mixture was vortexed for 30 s. The tube was placed in an ultrasonic bath (Grant Instruments XUB5 37 kHz, Grant Instruments, Royston, UK) at room temperature for 15 min to ensure complete dissolution. The solution was transferred quantitatively to a 10 mL volumetric flask using additional acidified methanol, and the volume was adjusted to the mark at 20 °C. The solution was mixed thoroughly and stored at 0 °C for 1 h to allow precipitation of any insoluble material. The solution was filtered through a 0.22 μm PTFE syringe filter (Millipore) into an HPLC vial. For samples with high bitter acid content, a 1:5 dilution was prepared by transferring 1.0 mL of the filtered solution to a 5 mL volumetric flask and diluting to volume with acidified methanol.

#### 4.5.2. HPLC Conditions

HPLC analysis was performed according to EBC 7.8 Method (Iso-α-, α- and β-Acids in Hop and Isomerised Hop Extracts by HPLC). Quantification of α-acids, β-acids, and iso-α-acids was performed using a Shimadzu Prominence liquid chromatography system (Shimadzu Corp., Kyoto, Japan) consisting of an LC-20AD pump, SIL-20AC autosampler, CTO-20AC column oven, and SPD-M20A photodiode array detector. Separation was achieved on a Kinetex 5 µm C18 100 Å column (250.0 mm × 4.6 mm, Phenomenex, Torrance, CA, USA) equipped with a SecurityGuard C18 pre-column (4.0 mm × 3.0 mm, Phenomenex). The mobile phase consisted of eluent A: HPLC-grade methanol (100%) and eluent B: Methanol (750 mL) + water (240 mL) + orthophosphoric acid (85%, 10 mL). The gradient program was 0–15 min, 0–100% B; 15–25 min, 100% B; 25–30 min, 100–0% B. The flow rate was 1.0 mL/min, column temperature was 40 °C, injection volume was 10 µL, and detection wavelengths were 270 nm (α-acids and β-acids) and 314 nm (iso-α-acids). Calibration curves for α-acids (ICE-4, 50–400 µg/mL), β-acids (ICE-4, 50–400 µg/mL), and iso-α-acids (ICS-I4, 25–200 µg/mL) were prepared at four validated concentration levels with coefficients of determination R^2^ ≥ 0.997. Although calibration standards were initially prepared at six concentration levels, the two lowest levels were excluded from the final regression model as back-calculated accuracy exceeded the ±20% acceptance criterion (ICH Q2(R1)) for at least one analyte at each of those levels. The limits of detection (LOD) and quantification (LOQ) were determined from the residual standard deviation of the calibration line and its slope (ICH Q2(R1)), with LOQ verified by back-calculated accuracy (±20% criterion): α-acids: LOD = 3.0–8.7 µg/mL, LOQ = 5.5–15.8 µg/mL; β-acids: LOD = 3.6–3.7 µg/mL, LOQ = 6.5–6.8 µg/mL; iso-α-acids: LOD = 1.5–6.1 µg/mL, LOQ = 2.1–8.8 µg/mL. All analyses were performed in triplicate.

### 4.6. Gas Chromatography–Mass Spectrometry (GC-MS) Analysis

Volatile compound profiles of unconcentrated cold ethanol extracts were analyzed by GC-MS using a GC-2010 Plus gas chromatograph coupled with a GCMS-QP2010 SE mass spectrometer (Shimadzu Corp.) equipped with an AOC-20i+s autosampler (Shimadzu Corp.) and an HP-5MS capillary column (30 m × 0.25 mm × 0.25 µm film thickness, Agilent Technologies, Santa Clara, CA, USA). Sample preparation: approximately 100 mg of extract was accurately weighed into a 10 mL volumetric flask; 20 µL of internal standard (IS) solution (2-undecanone, 50 µg/mL in cyclohexane) was added, and the flask was made up to volume with cyclohexane. After thorough mixing and complete dissolution of the extract, the sample was filtered through a 0.22 µm hydrophobic polytetrafluoroethylene (PTFE) syringe filter and transferred into a GC vial. Samples (1 µL) were injected in split mode (1:10) at an injector temperature of 250 °C. The oven temperature program was 50 °C (1 min hold), ramped at 8 °C/min to 200 °C (2 min hold), then ramped at 10 °C/min to 250 °C (1 min hold). Helium carrier gas flow rate was 1.0 mL/min. Mass spectra were acquired in electron ionization (EI) mode at 70 eV, scanning m/z 40–400. Compounds were identified by comparison of retention indices (RIs) and mass spectra with the NIST 17 library and literature data. Compounds were identified by comparison of mass spectra with the NIST 17 library; only compounds with a match similarity index ≥ 90% were retained for quantification. Compounds detected in blank runs (cyclohexane with 20 µL IS solution) and in IS-only control runs—both performed using the same syringes and vials as the cold ethanol extract runs—were excluded from the final compound list. Quantification was performed relative to 2-undecanone as the internal standard. All analyses were performed in triplicate.

### 4.7. Antimicrobial Susceptibility Testing

The minimum inhibitory concentrations (MICs) of hop extracts were determined in Mueller–Hinton broth (MHB; Pol-Aura) in accordance with the Clinical and Laboratory Standards Institute (CLSI) guidelines for broth microdilution susceptibility testing (CLSI, 2024) [39]. The aqueous and ethanolic hop extracts were provided as dry preparations. Dry aqueous extracts were dissolved in phosphate-buffered saline (PBS), whereas dry ethanolic extracts were dissolved in 30% dimethyl sulfoxide (DMSO) to obtain stock solutions at a concentration of 30 mg/mL. The stock solutions were subsequently serially diluted two-fold in Mueller–Hinton broth (MHB) to obtain the required concentrations for the antimicrobial susceptibility assay. The final concentration of DMSO in the test samples did not exceed 15%. A solvent control containing 15% DMSO without hop extract was included to assess the potential effect of DMSO on bacterial viability. Bacterial viability in the DMSO control did not differ from that observed in the MHB control.

### 4.8. Statistical Analysis

Data are presented as mean ± standard deviation (SD) for HPLC and GC-MS analyses and as individual MIC values for antimicrobial susceptibility testing. Kruskal–Wallis H tests were performed to assess the effect of extraction method on α-acid, β-acid, and iso-α-acid content, with effect size estimated by epsilon squared (ε^2^). Additional statistical analyses were performed to investigate the relationship between extract composition, extraction procedure, and antibacterial activity. Associations between HPLC-quantified α-acid, β-acid, and iso-α-acid contents and MIC values were assessed using Spearman’s rank correlation coefficient (ρ). Non-detected (nd) compounds were not assigned an arbitrary quantitative value and were therefore treated as missing observations in the corresponding correlation analyses. Statistical significance was set at *p* < 0.05. To evaluate the effect of the extraction procedure, MIC values obtained for the five extraction methods applied to all four hop varieties (hot ethanol, cold ethanol, espresso/steam, hot water, and cold water) were compared using the Friedman test, with hop variety treated as the matched factor. Supercritical CO_2_ extract was excluded from this analysis because this extraction procedure was applied to only one hop variety. Clinical *E. coli* was not subjected to correlation or between-method statistical comparisons because all tested extracts showed an identical MIC value of 15 mg/mL.

Statistical analyses were performed using Python 3.9 (Python Software Foundation, Wilmington, DE, USA; https://www.python.org) with the following libraries: NumPy 1.21 (https://numpy.org), pandas 1.3 (https://pandas.pydata.org), SciPy 1.7 (https://scipy.org) for Kruskal–Wallis, Spearman correlation and Friedman test, and Matplotlib 3.4 (https://matplotlib.org) with Seaborn 0.11 (https://seaborn.pydata.org) for visualization.

## 5. Conclusions

The present study characterized the antibacterial activity of *H. lupulus* hop extracts across five extraction methods and four hop varieties. The main finding is that the extraction method is the main factor determining the extract composition and antibacterial efficacy. Ethanol-based and supercritical CO_2_ extractions yielded the highest concentrations of prenylated acyl phloroglucinols (α-acids and β-acids) and, consequently, the best antibacterial efficacy against Gram-positive bacteria.

Ethanol-based and supercritical CO_2_ extraction protocols were identified as optimal methods for producing hop extracts with antibacterial properties, providing a rational basis for their application as natural antimicrobial agents against clinically relevant Gram-positive pathogens, including MRSA.

Moreover, clinical MRSA strains showed increased susceptibility to the selected ethanol extracts, consistent with a mechanism of action distinct from β-lactam resistance pathways.

The fact that hop α- and β-acids operate through mechanisms distinct from established resistance pathways supports their potential as anti-MRSA agents. In contrast, the limited susceptibility of MDR uropathogenic *E. coli* to most hop extracts is likely attributable to the permeability barrier of the Gram-negative outer membrane and the activity of efflux pumps. Among the tested extracts, only the supercritical CO_2_ extract exhibited measurable anti-UPEC activity.

Together, these findings demonstrate a clear relationship between the extraction method, chemical composition, and antibacterial activity of hop extracts. They also provide a foundation for the development of hop-derived antibacterial agents against Gram-positive multidrug-resistant pathogens, including MRSA.

## Figures and Tables

**Table 1:** Extraction yields and HPLC-quantified compound content.

Variety	Extraction Method	Yield [mg]	Individual Hop Compounds [% *w*/*w*]							Total Compound Content [% *w*/*w*]		
			Cohumulone	N+Adhumulone	Colupulone	N+Adlupulone	Isocohumulone	Isohumulone	Isoadhumulone	α-Acids	β-Acids	Iso-α-Acids
Citra	Hot ethanol	1600	3.430 ± 0.077	25.297 ± 0.660	3.551 ± 0.105	3.966 ± 0.107	0.079 ± 0.001	0.240 ± 0.004	0.236 ± 0.004	28.730 ± 0.614	7.520 ± 0.185	0.560 ± 0.021
	Cold ethanol	2000	7.078 ± 0.229	26.345 ± 0.512	3.686 ± 0.107	4.110 ± 0.063	0.034 ± 0.001	0.395 ± 0.013	0.011 ± 0.001	33.420 ± 0.521	7.800 ± 0.210	0.440 ± 0.018
	Espresso/steam	2520	1.177 ± 0.022	2.682 ± 0.050	0.119 ± 0.003	0.143 ± 0.004	nd	0.050 ± 0.003	nd	3.860 ± 0.115	0.260 ± 0.012	0.050 ± 0.003
	Hot water	970	0.382 ± 0.008	0.897 ± 0.024	nd	nd	0.159 ± 0.003	0.209 ± 0.004	nd	1.280 ± 0.045	nd	0.370 ± 0.015
	Cold water	1046	0.263 ± 0.006	0.448 ± 0.011	nd	nd	nd	nd	nd	0.710 ± 0.032	nd	nd
Lubelski	Hot ethanol	1450	2.078 ± 0.064	6.241 ± 0.119	4.222 ± 0.107	5.880 ± 0.158	0.073 ± 0.001	0.142 ± 0.004	nd	8.320 ± 0.241	10.100 ± 0.305	0.210 ± 0.011
	Cold ethanol	2050	2.774 ± 0.051	8.396 ± 0.137	5.649 ± 0.192	7.739 ± 0.266	0.084 ± 0.003	0.149 ± 0.003	nd	11.170 ± 0.312	13.390 ± 0.412	0.230 ± 0.010
	Espresso/steam	2140	0.507 ± 0.009	1.027 ± 0.029	nd	nd	nd	nd	nd	1.530 ± 0.055	nd	nd
	Hot water	1190	0.168 ± 0.004	0.348 ± 0.006	0.088 ± 0.002	0.129 ± 0.002	0.091 ± 0.003	0.122 ± 0.002	nd	0.520 ± 0.021	0.220 ± 0.014	0.210 ± 0.008
	Cold water	963	0.170 ± 0.005	0.294 ± 0.006	nd	nd	nd	nd	nd	0.460 ± 0.018	nd	nd
Magnat	Hot ethanol	1700	5.771 ± 0.147	24.851 ± 0.644	3.416 ± 0.064	4.401 ± 0.151	nd	nd	0.380 ± 0.016	30.620 ± 0.812	7.820 ± 0.225	0.380 ± 0.016
	Cold ethanol	2050	2.995 ± 0.101	12.894 ± 0.424	1.718 ± 0.046	2.206 ± 0.074	nd	nd	0.070 ± 0.004	15.890 ± 0.410	3.920 ± 0.105	0.070 ± 0.004
	Espresso/steam	1465	1.127 ± 0.021	2.485 ± 0.040	nd	nd	nd	nd	nd	3.610 ± 0.102	nd	nd
	Hot water	1170	5.771 ± 0.124	24.851 ± 0.566	3.416 ± 0.070	4.401 ± 0.139	nd	nd	0.610 ± 0.025	2.200 ± 0.088	nd	0.610 ± 0.025
	Cold water	930	0.466 ± 0.010	1.297 ± 0.034	nd	nd	nd	nd	0.050 ± 0.002	1.760 ± 0.074	nd	0.050 ± 0.002
Marynka	Hot ethanol	1500	3.738 ± 0.116	12.486 ± 0.206	3.402 ± 0.118	3.660 ± 0.111	0.126 ± 0.002	0.243 ± 0.004	nd	16.220 ± 0.450	7.060 ± 0.198	0.370 ± 0.017
	Cold ethanol	1680	4.524 ± 0.142	15.135 ± 0.441	4.090 ± 0.121	4.371 ± 0.133	0.094 ± 0.002	0.141 ± 0.003	0.010 ± 0.000	19.660 ± 0.515	8.460 ± 0.254	0.240 ± 0.012
	Espresso/steam	1800	0.700 ± 0.023	1.256 ± 0.034	nd	nd	nd	nd	nd	1.960 ± 0.062	nd	nd
	Hot water	1110	0.221 ± 0.005	0.446 ± 0.007	nd	nd	0.191 ± 0.004	0.230 ± 0.005	nd	0.670 ± 0.025	nd	0.420 ± 0.018
	Cold water	919	0.189 ± 0.006	0.300 ± 0.008	nd	nd	nd	nd	nd	0.490 ± 0.015	nd	nd
	Supercritical CO_2_	ND	9.683 ± 0.317	33.795 ± 0.826	6.003 ± 0.104	9.017 ± 0.264	0.114 ± 0.003	nd	0.096 ± 0.007	43.470 ± 1.120	15.020 ± 0.485	0.210 ± 0.011

Abbreviations: ND: no data; nd: not detected.

**Table 2:** GC-MS analysis of volatile compounds in unconcentrated cold-ethanol hop extracts.

Compound	RT (min)	KI	SI [%]	Molecular Weight-Corrected 2-Undecanone Equivalents [µg/mL]			
				Citra	Lubelski	Magnat	Marynka
β-Myrcene	6.801	965	96	1019.71 ± 97.11	282.70 ± 12.34	1154.99 ± 31.23	845.03 ± 14.80
D-Limonene	7.575	1012	95	9.47 ± 0.62	nd	nd	nd
Linalool	8.932	1094	95	18.25 ± 1.46	13.74 ± 1.47	23.52 ± 1.87	11.25 ± 0.87
Nerol	11.717	1262	93	10.18 ± 1.61	nd	nd	nd
Methyl trans-geranate	12.960	1337	94	67.79 ± 8.15	nd	9.70 ± 1.23	29.15 ± 0.84
β-Caryophyllene	14.690	1441	95	174.20 ± 9.49	31.67 ± 2.47	90.11 ± 3.81	154.96 ± 4.38
cis-α-Bergamotene	14.875	1453	97	nd	nd	14.76 ± 0.40	27.70 ± 1.39
(E)-β-Farnesene	15.154	1469	95	nd	97.87 ± 5.56	136.70 ± 4.65	550.00 ± 9.84
α-Humulene	15.268	1476	97	141.30 ± 15.14	99.31 ± 3.75	303.36 ± 8.91	249.99 ± 4.36
γ-Murolene	15.545	1493	95	10.93 ± 4.77	nd	nd	10.12 ± 1.09
trans-α-Bergamotene	15.753	1506	95	4.89 ± 2.46	nd	10.60 ± 0.87	nd
β-Selinene	15.794	1508	94	27.85 ± 2.56	nd	nd	12.99 ± 1.02
α-Selinene	15.899	1514	94	27.82 ± 3.03	nd	nd	14.57 ± 0.98
α-Farnesene	15.966	1518	95	19.15 ± 1.28	nd	10.54 ± 0.85	nd
γ-Cadinene	16.147	1529	96	9.20 ± 0.69	7.13 ± 0.46	10.89 ± 0.84	11.33 ± 0.56
β-Cadinene	16.214	1533	94	12.26 ± 1.34	nd	11.84 ± 0.69	15.44 ± 0.64
Humulenol II	17.964	1639	96	nd	6.52 ± 1.23	nd	nd
Z-5,17-Octadecadien-1-yl acetate	18.259	1657	96	11.36 ± 1.18	11.12 ± 2.28	nd	nd
Neointermedeol	18.324	1661	94	2.03 ± 3.52	nd	nd	nd
Dehydrocohumulinic acid	18.968	1700	93	33.70 ± 2.82	39.83 ± 9.93	nd	34.99 ± 6.39

Values are presented as means ± SD (*n* = 3). Concentrations are semi-quantitative, calculated relative to 2-undecanone and corrected for individual molecular weights. Only compounds with a NIST 17 library similarity index SI ≥ 90% are reported; compounds detected in blank (neat cyclohexane) or internal standard-only control runs were excluded. Abbreviations: RT: retention time; KI: Kovàts retention index; SI: NIST 17 library similarity index [%]; nd: not detected.

**Table 3:** Antimicrobial activity of hop extracts.

Variety	Extraction Method	Minimum Inhibitory Concentration (MIC) [mg/mL]			
		*E. coli* ATCC 25922	Clinical *E. coli*	*S. aureus* ATCC 29213	Clinical *S. aureus* MRSA
Citra	Hot ethanol	4.000	15.000	0.125	0.250
	Cold ethanol	4.000	15.000	0.125	0.125
	Espresso/steam	15.000	15.000	2.000	1.000
	Hot water	15.000	15.000	4.000	2.000
	Cold water	15.000	15.000	8.000	15.000
Lubelski	Hot ethanol	4.000	15.000	0.125	0.016
	Cold ethanol	4.000	15.000	0.125	0.250
	Espresso/steam	15.000	15.000	4.000	4.000
	Hot water	15.000	15.000	10.000	15.000
	Cold water	15.000	15.000	10.000	15.000
Magnat	Hot ethanol	4.000	15.000	0.250	0.250
	Cold ethanol	4.000	15.000	0.250	0.250
	Espresso/steam	15.000	15.000	2.000	2.000
	Hot water	15.000	15.000	4.000	4.000
	Cold water	15.000	15.000	4.000	15.000
Marynka	Hot ethanol	4.000	15.000	0.250	0.250
	Cold ethanol	4.000	15.000	0.250	0.016
	Espresso/steam	15.000	15.000	4.000	2.000
	Hot water	15.000	15.000	6.000	6.000
	Cold water	15.000	15.000	10.000	15.000
	Supercritical CO_2_	4.000	6.000	0.125	0.032

**Table 4:** Differences between extraction method and MIC value.

Bacterial Strain	Friedman χ^2^	*p*-Value
*E. coli* ATCC 25922	16.000	**0.003**
Clinical *E. coli*	*NA*	*NA*
*S. aureus* ATCC 29213	15.784	**0.003**
Clinical MRSA	15.282	**0.004**

Abbreviations: *NA*, not applicable because all extracts used against clinical *E. coli* had the same MIC value (15 mg/mL), resulting in no variance. Statistical significance was set at *p* < 0.05.

## Data Availability

The original contributions presented in this study are included in the article. Further inquiries can be directed to the corresponding author.
